# Positron Emission Tomography–Magnetic Resonance Imaging, a New Hybrid Imaging Modality for Dentomaxillofacial Malignancies—A Systematic Review

**DOI:** 10.3390/diagnostics15060654

**Published:** 2025-03-07

**Authors:** Anastasia Mitsea, Nikolaos Christoloukas, Spyridoula Koutsipetsidou, Periklis Papavasileiou, Georgia Oikonomou, Christos Angelopoulos

**Affiliations:** 1 Department of Oral Diagnosis & Radiology, School of Dentistry, National and Kapodistrian University of Athens, 2 Thivon Str., 11527 Athens, Greece; 2 Biomedical Sciences, Division of Radiology and Radiotherapy, University of West Attica, 28 Agiou Spiridonos Str., 12243 Athens, Greece

**Keywords:** PET MRI, head and neck tumors, malignancies, oral cancer

## Abstract

**Background/Objectives**: Emerging hybrid imaging modalities, like Positron Emission Tomography/Computed Tomography (PET/CT) and Positron Emission Tomography/Magnetic Resonance Imaging (PET/MRI), are useful for assessing head and neck cancer (HNC) and its prognosis during follow-up. PET/MRI systems enable simultaneous PET and MRI scans within a single session. These combined PET/MRI scanners merge MRI’s better soft tissue contrast and the molecular metabolic information offered by PET. Aim: To review scientific articles on the use of hybrid PET/MRI techniques in diagnosing dentomaxillofacial malignancies. **Method**: The available literature on the use of PET/MRI for the diagnosis of dentomaxillofacial malignancies in four online databases (Scopus, PubMed, Web of Science, and the Cochrane Library) was searched. Eligible for this review were original full-text articles on PET/MRI imaging, published between January 2010 and November 2024, based on experimental or clinical research involving humans. **Results**: Out of the 783 articles retrieved, only twelve articles were included in this systematic review. Nearly half of the articles (5 out of 12) concluded that PET/MRI is superior to PET, MRI, and PET/CT imaging in relation to defining malignancies’ size. Six articles found no statistically significant results and the diagnostic accuracy presented was similar in PET/MRI versus MRI and PET/CT images. Regarding the overall risk of bias, most articles had a moderate risk. **Conclusions**: The use of PET/MRI in HNC cases provides a more accurate diagnosis regarding dimensions of the tumor and thus a more accurate surgical approach if needed. Further prospective studies on a larger cohort of patients are required to obtain more accurate results on the application of hybrid PET/MRI.

## 1. Introduction

Staging head and neck cancer (HNC) involves clinical examination and imaging to determine various factors, including tumor size, extent, lymph node involvement, detailed spread (as bone infiltration and perineural infiltration), and the potential presence of distant metastasis [1]. The anatomical complexity of the head and neck supports fundamental functions and determines facial morphology. Consequently, precisely detecting primary and recurrent cancers in this area is essential for effective treatment planning that retains as much functionality and morphology as feasible [2]. Head and neck cancer (HNC) is the sixth most common cancer, affecting millions of people globally [3]. MRI and CT offer accurate information about anatomical structures concerning HNC due to their high resolution. They are also the principal imaging procedures utilized for T staging. Nevertheless, these imaging approaches have weaknesses, especially in evaluating involvement of nodes in the neck and recurrence of the disease [4,5,6,7]. One of the important limitations of CT concerns its low soft tissue contrast, which limits the distinction of various soft tissues. On the contrary, MRI is the appropriate imaging modality for evaluation of the extent of the malignancy into soft tissues [8]. Despite their limitations, CT and MRI are most frequently employed methods for T staging. CT, due to its low soft tissue contrast, cannot identify soft tissues, whereas MRI is a critical imaging method for detecting tumor expansion into soft tissues [8]. However, CT presents problems in recognizing the extension into the mandible and frequently overestimates the cortical invasion [9]. However, MRI offers several advantages, such as increased soft tissue contrast, particularly in complicated anatomical structures, and the ability to detect bone marrow malignancies in craniofacial bones. Furthermore, MRI offers inherent advantages, such as no radiation exposure and fewer metallic artifacts from dental prostheses [10,11,12,13].

^18^F-fluorodeoxyglucose (FDG) positron emission tomography (PET)/CT is widely utilized for staging, particularly for evaluating N (nodal) or M (metastatic) disease in head and neck cancers [14,15]. PET has been proven to enhance the sensitivity of lymph node staging compared to relying solely on morphological imaging [16]. By employing 18F-FDG, the diagnostic accuracy for lymph node staging can be significantly improved [17]. Moreover, PET/CT is particularly effective in identifying osseous lesions, making it an excellent diagnostic imaging method for detecting bone involvement, such as radio-osteonecrosis and metastatic disease. Several studies have also demonstrated that PET/CT outperforms PET/MRI in the detection and evaluation of notable bone lesions [18,19].

Furthermore, PET/CT has proven essential for the T staging of tumors that spread to difficult-to-access locations or to osseous structures because it aids with assessing infiltration of anatomical landmarks such as bone and bigger vessels [20]. Nonetheless, using low-dose CT in PET/CT presents limitations, such as restricted contrast resolution and occasional streak artifacts from dentures or inlays, which may cause difficulties in identifying FDG-avid lesions. Furthermore, the ability to identify cystic/necrotic lymph nodes and evaluate infiltration of adjacent structures, particularly perineural spread, is limited [21]. Consequently, it is feasible to combine both these approaches for clinical T staging of oral or oropharyngeal cancer cases with possible mandibular involvement. Software-based image fusion from sole MRI and PET systems (fused PET/MRI) has been reported to be useful in evaluating head and neck cancers [22,23,24]. Hybrid PET/MRI systems have been generated and are currently available for clinical use. They allow simultaneous collection of PET and MRI datasets during a single scan [25]. These PET/MRI scanners merge MRI’s strong soft tissue contrast with PET’s molecular and metabolic data. The development of reliable PET/MR imaging protocols for head and neck cancer staging is outlined in previous reports [26,27]. Using 18F-FDG, integrated PET/MRI enables simultaneous PET and MRI scans with superior soft tissue contrast, multi-layer image capture, and functional imaging features. This approach presented superior diagnostic accuracy for assessing the tumor’s local invasion, and identification of lymph nodes as well as metastatic disease distantly in head and neck cancer patients [10,28,29]. PET/MRI shows potential as an asset for patients with head and neck cancer and probable dementia [30]. Emerging hybrid imaging techniques, such as PET/CT and PET/MRI, are proving to be valuable tools for assessing HNSCC tumors in terms of prognosis and follow-up [31]. 18F-FDG-PET is thought to be superior for diagnosing osteomyelitis due to its improved spatial resolution and specificity. It effectively detects infected bone by capturing glucose transporters corresponding to inflammatory cells, microorganisms, and granulation tissue [32,33,34]. However, MRI has limitations in distinguishing edema and infection, and metal implants can affect diagnostic accuracy [35]. There is no single universally accepted method (like a gold-standard imaging modality), for all dentomaxillofacial malignancies. However, MRI is often considered the preferred imaging technique due to its superior soft tissue contrast and ability to assess tumor extent. Additionally, PET/CT is widely used for staging, detecting metastases, and treatment planning. Our review focuses on PET/MRI, which combines the advantages of both modalities, offering high soft tissue contrast with functional metabolic assessment [18,19,25,28,29,32,33,34,35].

Therefore, the objective of this systematic review is to evaluate the scientific literature published in the past decade regarding the use of PET/MRI hybrid imaging in diagnosing dentomaxillofacial malignancies. These malignancies may be located across the entire maxillofacial region, including the oral cavity, oropharynx, nasopharynx, larynx, sinuses, and salivary glands.

## 2. Materials and Methods

### 2.1. Design

The available literature on the use of a hybrid PET/MRI imaging approach for the diagnosis of dentomaxillofacial malignancies was evaluated. The review followed the PRISMA statement standards, as established by Page et al. [36].

### 2.2. Eligibility Criteria

Eligibility and exclusion criteria were applied when selecting the articles.

#### 2.2.1. Types of Outcome Measure

The study team thoroughly collected key details from each document using specially designed data collection forms. The features documented included sample size, ethnicity, gender distribution, age range, software used, PET/CT and/or PET/MRI units used, contrast enhancement, malignancy kind, and tumor site [36].

#### 2.2.2. Study Design

This systematic review exclusively focused on full-text original articles concerning PET/MRI hybrid imaging techniques on humans released in English. Experimental studies that fulfilled the systematic review criteria were also included, with no restrictions based on subject age, gender, or ethnic composition.

#### 2.2.3. Inclusion Criteria

Eligible for this review were original full-text articles on hybrid PET/MRI imaging techniques, published between January 2010 and November 2024, based on experimental or clinical research involving humans.

#### 2.2.4. Exclusion Criteria

Articles that belonged to the following categories were excluded: personal opinions, author debates, letters to the editor, author responses, newsgroups papers, abstracts, editor summaries, congress abstracts, overview papers, books, or book chapters. Furthermore, articles concerning animal experiments, non-English papers, case reports, methodologically inconsistent studies, and systematic reviews were also excluded.

### 2.3. Information Sources

Two of the authors, N.C. and A.M., performed comprehensive research using 4 online databases: Scopus, PubMed, Web of Science, and the Cochrane Library.

### 2.4. Search Strategy

Based on MeSH terms (medical subject headings) and free-text synonyms, two of the authors, N.C. and A.M., performed systematic research of 4 online databases (Scopus, PubMed, Web of Science, and the Cochrane Library), considering papers published during the above-mentioned time frame. To follow a comprehensive search strategy, Boolean operators like AND and OR were used, combined with terms such as “Pet MRI”, “head and neck”, “tumors”, “malignancies”, and “oral cancer” in various combinations.

### 2.5. Study Selection

Throughout the article retrieval process, the authors consistently followed the selection and exclusion criteria. All duplicate studies detected among the retrieved papers were removed. There were two distinct phases followed during the article selection process. During the initial phase, two reviewers (N.C. and A.M.) meticulously and independently assessed the titles and abstracts gathered from all electronic databases. Disagreements on which articles to consider for a full-text evaluation were settled through discussion. If necessary, a final decision was reached after consulting with a third reviewer (C.A.). Next, both reviewers (N.C. and A.M.) independently reviewed the whole text of each article. Whenever there was any disagreement on whether an article should be included in the review, the ultimate decision was taken through discussion. If they were unable to reach a final decision, a third auditor (C.A.) was contacted until an agreement was reached. The justifications for each article’s rejection were documented.

### 2.6. Data Collection and Data Items

Subsequently, the same two reviewers, N.C. and A.M., independently collected data using a predefined and customized data extraction table. Extracted data were cross-checked. Discrepancies were resolved by carefully cross-checking the extracted data against the original articles to ensure accuracy and consistency. Specifically, they verified details such as the correct study size, and gender distribution and age range of the studies’ samples, ensuring that no misinterpretations occurred. They also reassessed the reported imaging modalities (PET/CT, PET/MRI), the use of contrast agents, tumor locations, and software employed, ensuring alignment with the study methodologies. If any inconsistencies were found, the reviewers discussed the discrepancies, re-evaluated the studies, and refined the extracted data accordingly. When consensus could not be reached, a third reviewer (C.A.) was consulted for final validation. The data extraction form covered the following aspects: (a) basic details (authors’ names, year of publication, journal, ethnicity); (b) participant characteristics (study size, gender distribution, age range); (c) essential content from each article (PET/CT and/or PET/MRI usage, use of contrast enhancement, cancer type, tumor location, software employed).

### 2.7. Risk of Bias Assessment in Included Studies

Applying the risk of bias in non-randomized studies of interventions tool (ROBINS-I), which was created for non-randomized trials and was outlined by Sterne et al., two authors (N.C. and K.S.) independently evaluated the quality of the included studies [37]. When differences in assessment occurred, the authors reached an agreement after in-depth conversations. If the two authors were unable to come to an agreement, the article was referred to a third author, A.M., for the final assessment of quality ratings. The two reviewers assessed the risk of bias using the ROBINS-I tool, evaluating each study based on key parameters: (1) confounding factors, (2) selection of participants, (3) classification of interventions, (4) deviations from intended interventions, (5) presence of missing data, (6) measurement of outcomes, and (7) selection of reported results. Each parameter was rated as low, moderate, serious, not applicable, or not informative. Discrepancies in assessment were resolved through discussion, and if necessary, a third reviewer (A.M.) provided the final judgment.

### 2.8. Effect Measures and Data Synthesis

This project aimed to systematically investigate the available literature and evaluate the scientific evidence published between January 2010 and November 2024 concerning the utility of hybrid PET/MRI techniques in diagnosing dentomaxillofacial malignancies. The variables identified in each article were authors’ names, year of publication, journal, sample characteristics (study size, gender distribution, age range), PET/CT and/or PET/MRI usage, use of contrast enhancement, cancer type, tumor location, and software employed.

## 3. Results

### 3.1. Description of Studies

**Figure 1:**
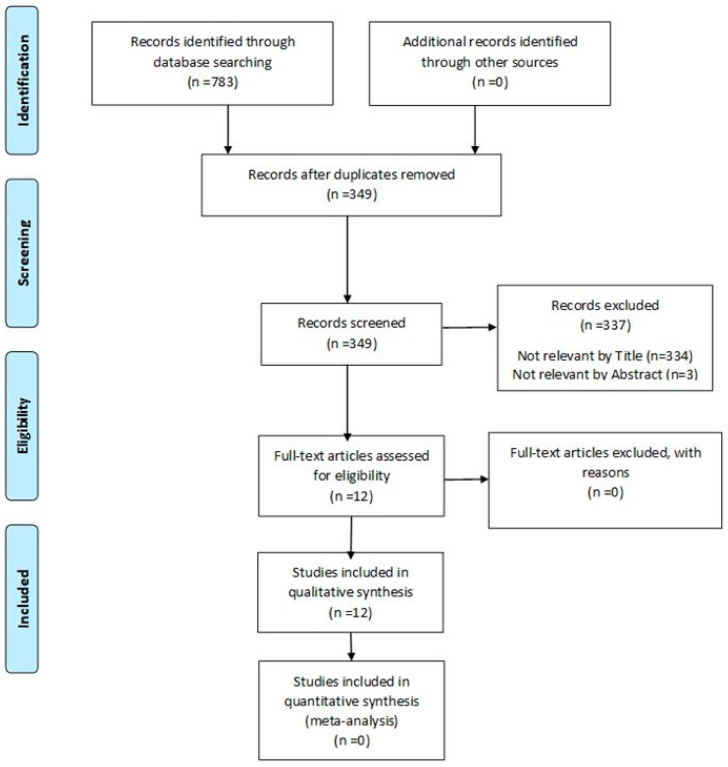
PRISMA flow diagram.

The results of the literature search, and the process followed to reject and select the articles included in this systematic review, are presented in a flow chart following the PRISMA statement guidelines (Figure 1). After the electronic and manual search of the databases, a total of 783 relevant articles were initially identified. After careful screening, duplicates were removed, leaving 349 articles. Articles that were not relevant, based on their title and abstract, were then rejected, and finally twelve articles met the selection and exclusion criteria and were included in the review. The twelve articles included in this systematic review were released between 2010 and 2024. Four articles included Germans as the demographic sample; three articles included Japanese populations; two articles included Polish; one article included Hungarian; one article included Swiss; and one article included a Korean population.

Table 1 provides details about the 417 patients (269 men and 148 women) who comprised the population sample in these 12 studies overall. The sample size of each study varied significantly, ranging from 108 to 6 patients [38,39]. The sample size included less than 50 patients in the majority of the articles studied. However, three studies conducted by Park et al. (2020) [39], Schaarschmidt et al. (2017) [40], and Queiroz et al. (2014) [41], reported results based on larger subject numbers, specifically 81, 87, and 108 participants, respectively. Due to the significant heterogeneity among these articles, conducting a meta-analysis was not feasible.

Of the twelve, only five articles assessed whether the PET/MRI hybrid imaging technique can be useful in the diagnosis of Head and Neck Squamous Cell Carcinoma (HNSCC) cases [31,42,45,46,48]. There were four studies on cancer of unknown primary location including recurrent tumors, lymph node metastases, and primary malignant neoplasms [39,40,41,44]. One article investigated tongue cancer, another one dealt with oral cancer, and the last one focused on an osteomyelitis case [38,43,47].

Nearly half of the articles (5 out of 12) concluded that PET/MRI is superior to PET, MRI, and PET/CT imaging in relation to defining malignancies’ size [39,42,44,45,47,48]. On the other hand, six articles found no statistically significant results and diagnostic accuracy was similar in PET/MRI versus MRI and PET/CT images [31,38,40,41,43,45]. 

### 3.2. Risk of Bias

The risk of bias was assessed for all twelve articles included in this systematic review as presented in Table 2. In terms of overall risk of bias, a moderate risk characterized most of the papers. Regarding the overall risk of bias, most articles presented a moderate risk. The designation of a moderate risk of bias in most articles was based on the evaluation of key parameters outlined in the ROBINS-I tool. Specifically, the overall risk was determined by the most serious score assigned to any of the assessed parameters, which collectively indicated a moderate risk across the studies.

## 4. Discussion

In this thorough review, an in-depth analysis of previous literature was conducted on the application of the PET/MRI hybrid imaging technique for diagnosing dentomaxillofacial malignancies, utilizing information from four different electronic databases. 18F-FDG PET enables the visualization of areas with increased glucose uptake, a characteristic feature of carcinomas [49]. As 18F-FDG PET images provide high contrast, but limited spatial resolution, they are often combined with morphological imaging methods such as CT or MRI [50]. A prospective study by Lonneux et al. (2010), demonstrated that the combination of 18F-FDG PET/CT, as opposed to CT alone, offers improved accuracy and higher sensitivity, particularly in the assessment of metastatic lymph nodes [51]. The advantages of PET/CT and MRI over CT alone have prompted investigations into the utility of images obtained from PET/MRI hybrid systems for target volume delineation. These benefits include enhanced diagnostic accuracy, potentially lower costs or reduced examination times, reduced radiation exposure, and the provision of additional information not obtainable through standard methods [41].

As suggested, volumetric metabolic parameters, for instance TLG (Total Lesion Glycolysis) and MTV (Metabolic Tumor Volume), might offer more thorough information concerning a tumor’s metabolism. According to earlier studies, TLG and MTV presented enhanced prognostic significance compared to SUVmax (Maximum Standardized Uptake Value). These studies also proved that increased tumor volume is connected to overall survival and lower local control rates [52,53]. Diffusion-weighted imaging–magnetic resonance imaging (DWI MRI) is widely employed for evaluating the motion of water molecules (Brownian motion) [54]. This non-invasive diagnostic technology dissects tissue biology by studying the motion of water molecules at a microscopic level [41]. Numerous studies have demonstrated the utility of DWI, represented as the apparent diffusion coefficient (ADC), across various clinical applications, ranging from interpreting the tumors’ microstructures to evaluating treatment responses [55,56]. Furthermore, DWI exhibited promising results in assessing the HPV status of patients with OPSCC (Oropharyngeal Squamous Cell Carcinoma) [57,58,59]. We did not consider these prognostically important parameters from the beginning because they emerged after completing the research methodology. Our original objective was to conduct a systematic review highlighting PET/MRI as an alternative imaging modality for maxillofacial malignancies. The exploration of volumetric metabolic parameters like TLG and MTV became relevant as we evaluated the findings from the included studies. The implications of volumetric metabolic parameters, such as TLG and MTV, in relation to imaging evaluation methods like PET/MRI and DWI are significant. TLG and MTV provide a more comprehensive understanding of tumor metabolism and aggressiveness compared to traditional metrics like SUVmax. In PET/MRI, these parameters enhanced the assessment of tumor burden and treatment response, aiding in prognosis and therapeutic decision-making. Similarly, in DWI, parameters like the apparent diffusion coefficient (ADC) can reflect tumor microenvironment changes and treatment efficacy, offering valuable insights into tumor biology. These parameters improved the accuracy of imaging evaluations and contribute to more personalized patient management [52,53,54,55,56,57,58,59].

Hayashi et al. (2019) [42] aimed to evaluate the efficacy of fused PET/MR images from different scanners in operation planning concerning oral/oropharyngeal malignancies with suspected mandibular invasion. The sample consisted of eleven patients (eight males and three females) who fulfilled certain inclusion criteria. RI and PET/CT exams were performed, and PET images were fused automatically. The PET/MR images were evaluated by two nuclear medicine physicians, and the results were confirmed histopathologically. Histopathological confirmation of invasion into the mandible and/or medial pterygoid muscle according to the PET/MR images was possible in 81% of the patients. The correlation coefficient of the MRI-estimated tumor size against histopathological measurements was moderate. Fused PET/MRI was superior to MRI and PET/CT in detecting mandibular and medial pterygoid muscle invasion, with certain limitations associated with the use of contrast-enhanced MRI and FDG PET/CT, a low specificity (40%) in detecting mandibular invasion, and false-negative findings in some cases concerning superficial cortical invasion. The value of PET/MRI was underscored in surgical treatment for oral/oropharyngeal cancer with suspected mandibular invasion [42].

Kanno et al. (2020) [43] evaluated the primary tumor extent in OTC (oral tongue cancer) based on the diagnostic performance of 18F-FDG PET/against ceMRI (contrast-enhanced MRI). Both scans were performed on 18 OTC patients in two weeks. Taking surgical pathology as a gold standard, tumor dimensions and depth of invasion (DOI) were visually measured on 18F-FDG PET/MRI and ceMRI. In two cases, artifacts prevented ceMRI from detecting tumors. Concerning tumor size, a significant difference was observed only between ceMRI and pathology. The accuracy of T status was lower in ceMRI (72%) than in ^18^F-FDG PET/MRI (89%). Small sample size, heterogeneity, and possible impacts of scan sequence and glucose levels on diagnostic accuracy are the main limitations of this study [43].

A study was carried out by Tsujikawa et al. (2020) [60] to determine if ZTE (Zero Echo Time) MRI could be utilized for jawbone imaging and to compare the diagnostic efficacy of 18F-FDG PET/MRI with ZTE-AC vs. PET/CT and PET/MRI with Dixon-AC in cases with OCC. In 13 consecutive patients with recently diagnosed OCC, a regional ^18^F-FDG PET/MRI and whole-body ^18^FFDG PET/CT were performed on the same day. Dixon-AC and ZTE-AC were applied to analyze PET images for both PET/CT and PET/MRI, including primary OCC tumors and metastatic CLNs (metastatic cervical lymph nodes). Maximum SUVs (SUVCT, SUVDixon, and SUVZTE) were calculated using a spherical region of interest. Both Dixon and ZTE-based SUVs were substantially related to SUVCT when primary OCC tumors were involved. Nevertheless, there was no differentiation between SUVDixon and SUVZTE. The CLN values for SUVDixon and SUVZTE were both very high. The comparison of Dixon and ZTE revealed a substantial positive connection between SUVDixon and SUVZTE regarding the primary OCC tumor. Moreover, there was barely any difference between SUVDixon and SUVZTE. In CLNs, the SUVDixon and SUVZTE scores were significantly greater than SUVCT. SUVDixon values were considerably higher than SUVZTE values. ZTE MRI scans provided good visualization of the jawbone and a low artifact level generated by artificial dentures. The restricted sample size, dependence on the highest SUVs for quantitative assessments, and the possibility of errors in CLN clinical diagnosis were some weaknesses of the study. Overall, ZTE MRI scans, particularly with ZTE-AC in 18F-FDG PET/MRI, have shown efficacy for decreasing tracer uptake underestimations due to Dixon-AC errors and improving PET quantitative outcomes in OCC patients [60].

Park et al. (2020) [39] evaluated the diagnostic accuracy and confidence levels of PET/MRI compared to individual PET or MRI usage in 73 consecutive patients with suspected head and neck malignancies. PET/MRI and MRI presented higher AUC values than PET alone for non-nodal lesions, while, concerning nodal lesions, PET/MRI presented a higher AUC than MRI. The concordance rate of PET/MRI was 82.8%, outperforming PET and MRI individually, particularly in the initial work-up and recurrence assessment. In conclusion, PET/MRI provides superior diagnostic accuracy and confidence in diagnosing head and neck malignancies [39].

Samołyk-Kogaczewska et al. (2020) [46] investigated the value of PET/MRI in presurgical staging of HNSCC (head and neck squamous cell carcinoma). According to PET/MRI, there was significant correlation between SUVmax and maximum tumor diameter. This was higher than the compatibility of PET/CT with histopathological (HP) results (in 67% of cases) and CT (in 56% of cases). Therefore, in HNSCC cases, PET/MRI seems more accurate for preoperative staging [46]. It should be mentioned that the studies contacted by Schaarschmidt et al. (2017) [40], Schaarschmidt et al. (2016) [45], Samołyk-Kogaczewska et al. (2019) [48], Loeffelbein et al. (2014) [44], and Queiroz et al. (2014) [61] did not demonstrate any advantages of PET/M concerning earlier detection of primary malignancies or a higher sensitivity, in comparison to PET, MRI, or PET/CT.

Loeffelbein et al. (2014) [44] attempted to evaluate the diagnostic accuracy of retrospective integration of PET-MRI regarding head and neck cancer staging compared to side-by-side analysis and single use of PET and MRI. The study sample included 33 subjects, who received preoperative examination with contrast-enhanced MRI and PET/CT. MRI and PET images, side-by-side, and retrospective PET-MRI fusion findings were assessed, and the accuracy of tumor and lymph node staging was evaluated using ROC analysis. These data were associated with histopathological findings. The authors did not observe any meaningful differences in terms of tumor identification. PET presented high reliability in cervical lymph node metastasis detection, higher than side-by-side analysis and PET-MRI fused images. These results aligned with another study by Ng et al. (2005) [62], who found that PET analysis was better at staging nodes versus MRI alone. A rising trend of the accuracy diagnostics across the single mode was also reported [62]. Additional lesions in T staging were detected; however, three malignant tumors were not visualized during simultaneous MRI and PET in side-by-side analysis. The predictive values of N staging were different, while the side-by-side analysis method presented higher values. The study presented limitations, such as the retrospective design, heterogeneity of patients, and the comparatively small sample [44].

Schaarschmidt et al. (2016) [45] conducted a similar study to Loeffelbein et al.’s (2014) [44], in which 18F-FDG PET/MR was used for the detection of primary local HNSCC tumors for initial staging and recurrent disease. In the current study, two observers reviewed T and N staging after reviewing MRI fused with 18FFDG PET/CT and MRI fused with 18F-FDG PET/MR scans. Regarding N staging in the initial staging group (12 patients), PET/MR had 75% accuracy, PET/CT had 59%, and MRI had 50%, while specificity was 71% for PET/MR, 77% for PET/CT, and 75% for MRI. None of the variables presented statistically significant differences. The accuracy of PET/MR was 72%, 72% for PET/CT, and 57% for MRI in the recurrence group (13 patients). There were no further significant differences between the two groups. The non-simultaneous performance of PET/MRI and PET/CT was a limitation of this study; future prospective studies should carry out scans concurrently [45].

Samołyk-Kogaczewska et al. (2019) [48] evaluated the accuracy of PET/MRI hybrid imaging in defining gross tumor volume (GTV) during radiotherapy planning for tongue carcinoma in ten patients with squamous cell carcinoma (SCC) of the tongue. Determining GTV metabolic activity included visual interpretation, and an automatic method based on a predefined SUV threshold. GTV-MRI was greater than GTV-CT in 80% of cases and GTV-PETvis in 40% of cases. The results of computer-aided manual delineation revealed small but statistically insignificant differences between MRI, PET, and CT. PET/MRI is advantageous compared to other imaging approaches as it improves SCC radiotherapy planning. SUVmax at 30% was established to be the most frequently matched mark for the contour of the primary tumor. This research evaluated PET/MRI’s diagnostic potential quite efficiently, although it was a retrospective study with small sample [48]. In Schaarschmidt et al.’s study (2017) [40], although they recognize the enhanced soft tissue contrast of PET/MRI, they reported that PET/MRI and PET/CT presented comparable accuracy in local tumor staging and lymph node assessment. Tracer uptake in the tonsils, pharynx, and thyroid gland can be frequently observed [63]. Rare instances of second unrelated malignancies require further investigation [64,65,66].

Schaarschmidt et al. (2017) [40] examined 81 oncologic patients who underwent contrast-enhanced 18F-FDG PET/CT and subsequent PET/MRI. Two reviewers independently assessed incidental tracer uptake from both examinations, revealing similar performance in characterizing incidental 18FFDG uptake in head and neck examinations. The study’s limitations included its retrospective nature, a small and diverse cohort, and the absence of histopathologic verification for each lesion [40].

Freihat et al. (2021) [31] considered 33 patients with oral primary oropharyngeal squamous cell carcinoma (OPSCC), to evaluate the prognostic efficacy of FDG PET and DWI imaging parameters (SUVmax, TLG, MTV) in predicting HPV status and prognosis after 6 months of therapy. The FDG values and HPV status did not present substantial differences. Patients with a higher N stage presented an increased SUVmax. The sole parameter that independently affected ADC alterations was HPV level. The limited sample, retrospective nature, single-center methodology, and standard FDG and DWI parameters employed in the absence of texture analysis were highlighted as the study’s drawbacks [31].

Queiroz et al. (2014) [41] evaluated the diagnostic accuracy of contrast-enhanced PET/MRI (cePET/MRI) in detecting recurrent head and neck cancer (HNC), comparing it with other modalities like contrast-enhanced CT (ceCT), contrast-enhanced MRI (ceMRI), and contrast-enhanced PET/CT (cePET/CT). A total of 87 adult patients were referred for clinical PET/CT to restage or follow up on various HNCs. Image analysis involved a trimodality setup with a focus on squamous cell carcinoma (SCC). Comparable diagnostic accuracy between cePET/MRI (91.5%) and cePET/CT (90.6%) was revealed. Both methods had similar artifacts, but their locations varied, with cePET/CT artifacts primarily in the suprahyoid area and cePET/MRI among supra and infrahyoid neck regions. In addition, both techniques helped to “resolve” inconclusive FDG findings by cePET/MRI in 11 cases and cePET/CT in 5. A total of 46 inflammatory and not-tumor-related additional pathologies were located by PET/CT and PET/MRI [41].

In 2022, Reinert et al. [38] examined the imaging features of OMJ (osteomyelitis of the jaw) by comparing 18F-fluoride PET, 18F-FDG PET, CT, and MRI images. The study’s sample was six symptomatic women, with infectious and noninfectious chronic types of OMJ. OMJ, an inflammatory bone disease, can affect the medullary cavity, cortical bone, periosteum, and soft tissues, categorized into acute, secondary chronic, and primary chronic OMJ [67]. Clinical features and previous treatments were evaluated to set the diagnosis [68,69]. In four patients, 18F-FDG PET/MRI and 18F-fluoride-PET/CT were performed, while two received MRI in conjunction with 18F-fluoride PET/CT or 18F-FDG PET/MRI. The quantitative and qualitative evaluation performed by a consensus panel revealed that, in 80%, the extent of impacted areas with enhanced PET tracer uptake in 18F-FDG PET was smaller than CT and MRI results. Only one subject presented equal distribution. The limited sample highlighted the need for extensive, prospective studies [38].

Of the twelve studies included in this review, half confirmed the advantages of PET/MRI over CT, MRI, and PET/CT. Combined highly contrast-enhanced MRI and ^18^F-FDG PET/CT applied for TNM staging may be replaced by 18F-FDG PET/MRI since it offers high soft tissue contrast and functional pictures that indicate tumor glycolytic activity [60].

According to Hayashi et al. (2019), in oral or oropharyngeal cancer patients, PET/CT or fused PET/MRI outperformed simple MRI in determining tumor size [42]. Fused PET/MRI was also more efficient than MRI in detecting pterygoid muscle invasion and was less impacted by dental artefacts [20,25]. A study by Huang et al. (2011) [24] noticed that fused PET/MRI had the highest correlation coefficient for measuring tumor dimensions and the highest sensitivity (90%) and specificity (91%) for evaluating focal invasion in advanced oral squamous cell carcinoma [24].

Findings from other studies have confirmed the superiority of PET/MRI in various aspects of head and neck cancer imaging and reinforced the potential benefits of PET/MRI in clinical practice and research. T staging performed with PET/MRI achieved an accuracy of 75% in cases of head and neck cancer, compared to 59% with PET/CT and 50% with MRI alone [45]. A study by Chen et al. (2018) revealed that PET/CT defined the dimensions of primary nasopharyngeal tumors more accurately compared to PET/CT [29]. The same outcomes were supported by other studies, which fused PET and MR imaging over PET/CT in terms of providing enhanced accuracy in T staging in cases with head and neck malignancies [2].

Tsujikawa et al. (2018) [60] demonstrated that FDG PET/MRI, with its superior soft tissue contrast, multiplanar image acquisition, and functional imaging capabilities, is valuable for evaluating the stages of oral cancers and assessing oropharyngeal cancer patients based on the new AJCC TNM staging system [60]. Platzek et al. (2013) [70] found that PET/MRI could detect 64% more metastatic lymph nodes, partly since PET/MRI scans were performed following PET/CT scans without the need for additional FDG injection, and the time interval needed between the FDG injection and PET/MRI contributed to increased FDG uptake by the lymph nodes [70].

According to Geraldine et al.’s (2017) study, PET/MRI staging of primary tumors of H and N cancers demonstrated noticeably higher diagnostic accuracy than cervical node staging, with 94% of primary tumors and 82% of regional nodes accurately staged in comparison to histological results [71]. These are in concordance with Platzek et al.’s (2013) [70] findings, which indicated that PET/MRI located more primary tumors than MRI or PET alone [70]. Another study conducted by Lee et al. (2014) [72] mentioned that PET/MRI can identify more primary tumor lesions than MRI, but a statistically significant evaluation has not been performed [72].

Previous research on the diagnostic accuracy of integrated 18F-FDG PET/MRI for the T staging of OCC has been published; however, these studies have limitations including use of the seventh edition of the AJCC cancer staging system, small sample sizes, and mixed types of malignancies [45,72,73,74]. Just like any imaging method, PET/MRI has limitations that one should consider. In histologically diagnosed benign lesions, PET/MRI could give false-negative results, particularly in small tumors [20]. However, in malignant concordant cases, PET/MRI can also give false-positive results due to inflammatory nodes or lymphoid hyperplasia, which appear bigger on MRI and may be hypermetabolic on PET [75]. To this extent, discordant cases were shown to have better diagnostic accuracy with MRI in terms of necrotic tumors, perineural spread, and low metabolic activity of tumor, such as low-grade lymphoma [76,77].

Furthermore, the PET/MRI system is expected to play a more significant role in future multiparametric comparative analyses that combine various pieces of functional information from advanced MRI techniques (e.g., diffusion-weighted imaging, perfusion-weighted imaging, MR spectroscopy, etc.) with metabolic information from PET [78].

In this study, ceMRI and 18F-FDG PET/MRI were observed to overestimate the DOI (depth of invasion) of tumors around 5 mm or smaller size. The results presented by Baba et al. (2020) [79] and Murakami et al. (2019) [80], which noted comparable overestimations when utilizing ceMRI, were in accordance with this [79,80]. Ahmed et al. (2010) [81], used T1-weighted post-contrast MRI sequences to delineate GTVs (gross tumor volumes) in cases of tumors at the tongue’s base and reported overestimations, supporting our findings [81]. Delouya et al. (2011) [82] found that, in most cases, GTVs of primary tumors were smaller when delineated on 18F-FDG PET/CT and CT alone, consistent with the overestimation observed in our study [82]. Hence, other authors have also mentioned this pattern, as reported by Leclerc et al. (2015) [83] and Guido et al. (2009) [84].

In contrast, Kanda et al. (2013) [23] examined 30 patients with oral cavity and hypopharynx cancer and reported that fused 18F-FDG PET/MRI had 87% accuracy for T status, slightly lower than MRI, which presented 90% accuracy. Both modalities were found to be significantly superior to 18F-FDG PET/CT, which presented 67%. Accordingly, in certain circumstances, MRI and PET/MRI might prove useful in precisely determining T status [23]. This has been documented in primary staging in a number of malignancies like pediatric, endometrial, and pancreatic tumors [85,86,87,88]. Additionally, PET/MRI demonstrated superior sensitivity compared to MRI alone when evaluating follow-up patients with head and neck malignancies (HNCs), emphasizing the possible benefits of PET/MRI in particular clinical situations [22].

Conversely, Loeffelbein et al. (2014) [44] retrospectively compared the diagnostic accuracy of fused PET/MRI and side-by-side analysis (i.e., simultaneous review of PET/CT and contrast-enhanced MRI data on two adjacent screens) for local and nodal staging of head and neck cancer. They found that these imaging modalities did not significantly differ from one another [44]. Likewise, the three modalities (MRI, 18F-FDG PET/CT, and 18FFDG PET/MRI) did not significantly differ from one another, according to Schaarschmidt et al. (2016). These findings were contrary to earlier research, which had repeatedly demonstrated the enhanced diagnostic accuracy of PET/CT over CT or MRI in a range of oncologic cases [87,88]. Due to the highly accurate localization of 18F-FDG tracer uptake on the matching and recorded CT image, patients have profited from the exact diagnosis of nodal tumor spread [89]. It was also proved that the morphologic dataset helped explain tracer uptake in atypical regions that are difficult to distinguish as functional or nonmalignant based solely on PET scans [90]. The main limitations of our study arise from the absence of similar systematic reviews for comparison. Additionally, our research was restricted to studies published in the English language.

## 5. Conclusions

New hybrid imaging modalities, such as PET/CT or PET/MRI, have emerged as useful technologies to evaluate HNC tumors in terms of prognosis and follow-up. Although some studies did not report higher diagnostic accuracy in diagnosing tumors (locoregional tumor) and cancer recurrence diagnosis compared to PET/CT and MRI, it is certain that the PET/MRI combination provides more information than other standard imaging studies, which may increase the accuracy in delineating the specific target tumor. Further prospective studies in a larger cohort of patients are required to obtain more accurate results on the application of hybrid PET/MRI. PET/MRI appears to be a promising modality for patients with head and neck malignancies, as well as for patients with suspected dementia. Consequently, nuclear medicine specialists and head and neck surgeons may be able to provide a more accurate diagnosis as far as it concerns the size and dimensions of a tumor and thus a more accurate surgical approach if needed.

## Figures and Tables

**Table 1:** Characteristics of studies included in the systematic review.

Authors	Publication Year	Title	Journal	Age Range	Software	Gender	Sample Size	PET CT	PET MRI	Contrast Enhance-ment	Type of Cancer	Tumor Region	Tesla	Origin	SUV PET
Hayashi et al. [42].	2019	Clinical Value of Fused PET/MRI for Surgical Planning in Patients with Oral/ Oropharyngeal Carcinoma	Laryngo- scope	54–85 yrs	GE Advantage Workstation 4.5	8 M 3 F	11 participants	Discovery 600 Motion System	1.5 T Avanto Siemens	lack of contrast- enhancement	Squamous cell carcinoma of mandible, lower gingiva, buccal mucosa	mandible, medial pterygoid muscle	1.5 T	Japan	Not mentioned
Kanno et al. [43].	2020	Comparison of diagnostic accuracy between [18F] FDG PET/MRI and contrast-enhanced MRI in T staging for oral tongue cancer	Annals of Nuclear Medicine	38–88 yrs	GE Advantage Workstation 4.5	7 M 11 F	18 participants	Biograph mCT Flow (Siemens)	3T PET/MR GE Healthcare	Gadolinium- diethylene- triamine	oral tongue cancer	tongue	3 T	Japan	0–10
Loeffelbein et al. [44].	2014	Diagnostic value of retrospective PET-MRI fusion in head-and-neck cancer	BMC Cancer	27–72 yrs	Not Mentioned	21 M 12 F	33 participants	Siemens Biograph Sensation 64 PET/CT	Magnetom Verio 3T Siemens	Contrast agent (I.V) (Imeron 300, 80–120 mL)	head and neck region (primary malignant neoplasm, recurrent tumor disease, lymph node metastasis)	oropharynx, tongue, the mouth floor, hypopharynx, buccal mucosa, vallecula, tonsil, salivary gland	3 T	Germany	SUV of five (5)
Schaarschmidt et al. [45].	2015	Locoregional tumour evaluation of squamous cell carcinoma in the head and neck area: a comparison between MRI, PET/CT and Integrated PET/MRI	Eur J Nucl Med Mol Imaging	56.5 ± 8.6 yrs	OsiriX Workstation (Pixmeo SARL, Bernex, Switzerland)	23 M 2 F	25 participants	Biograph mCT (Siemens)	Biograph mMR Siemens	Contrast agent (I.V) (Ultravist, 60 mL)	head and neck squamous cell carcinoma (HNSCC)	head and neck squamous cell carcinoma (HNSCC)	N/M	Germany	Primary 10.5 ± 3.0 (PET/CT), 13.6 ± 6.1 (PET/MR), Recurrent 8.0 ± 3.8 (PET/CT), 8.6 ± 4.4 (PET/MR)
Samołyk- Kogaczewska et al. [46].	2019	PET/MRI-guided GTV delineation during radiotherapy planning in patients with squamous cell carcinoma of the tongue	Strahlenther Onkol	36–66 yrs	Siemens syngo.via VB10B	5 M 5 F	10 participants	320-slices CT scanner Aquilion ON	3T Siemens Biograph mMRI Siemens	Contrast agent (I.V) (Ultravist 300, 1 mL/kg)	squamous cell carcinoma of tongue (SCC)	squamous cell carcinoma of tongue	3T	Poland	SUV_max_ 4.66–20.6 SUV_mean_ 2.61–12.4
Tsujikawa et al. [47].	2020	Zero Echo Time-Based PET/MRI Attenuation Correction in Patients With Oral Cavity Cancer	Clinical Nuclear Medicine	45–90 yrs	Not Mentioned	8 M 5 F	13 participants	Biograph mCT Flow Siemens	3T Signa PET/MR Healthcare	NA	oral cavity cancer	tongue, mandibular gingival, maxillary gingival, buccal mucosa, oral floor	3T	Japan	PRIMARY OCC: 14.4 ± 8.0 SUV(CT) 14.5 ± 8.6 SUV(Dixon) 15.6 ± 8.8 SUV(ZTE) CLNs 6.3 ± 3.0 SUV(CT) 8.0 ± 4.0 SUV(Dixon) 7.6 ± 3.9 SUV(ZTE)
Schaarschmidt et al. [40].	2017	Is integrated 18F-FDG PET/MRI superior to 18F-FDG PET/CT in the differentiation of incidental tracer uptake in the head and neck area?	Diagn Interv Radiol	54.4 ± 15 yrs	OsiriX (Pixmeo SARL).	41 M 40 F	81 participants	Biograph mCTTM Siemens	Magnetom Verio 3 T Siemens	Ultravist Bayer Healthcare (I.V, 70 mL)	head and neck region (primary malignant neoplasm, recurrent tumour disease, lymph node metastasis)	salivary glands, oral and nasal cavity, thyroid, unknown primary tumor	Not men- tioned	Germany	mean SUV_max_, 5.0 ± 1.9 (PET/CT) 5.9 ± 3.0 (PET/MRI)
Samołyk- Kogaczewska et al. [48].	2020	Usefulness of Hybrid PET/MRI in Clinical Evaluation of Head and Neck Cancer Patients	Cancers	36–74 yrs	Not Mentioned	9 M 12 F	21 participants	Aquilion CX	Siemens Healthcare GmbH	Ultravist (300, 1 mL/kg)	squamous cell carcinoma	tongue, buccal mucosa, lower gingiva, base tongue maxillary alveolar ridge, submandibular salivary gland	3 T	Poland	NI
Freihat et al. [31].	2021	Pre-treatment PET/MRI based FDG and DWI imaging Parameters for predicting HPV status and tumor response to chemoradiotherapy in primary oropharyngeal squamous cell carcinoma (OPSCC)	Oral Oncology	61.4 ± 0.7 yrs	Siemens Syngo Via (20VB)	23 M 19 F	33 participants	NA	Biograph mMR Siemens	Gadovist Bayer Healthcare	oro- pharyngeal squamous cell carcinoma	HPV	3 T	Hungary	Primary tumors: 12.61 ± 0.5
Queiroz et al. [41].	2014	PET/MRI and PET/CT in follow-up of head and neck cancer patients	Eur J Nucl Med Mol Imaging	24–90 yrs	Not Mentioned	68 M 19 F	87 participants	GE Healthcare	GE Healthcare	Visipaque 320 (I.V, 70 mL), GE Healthcare (0.2 mL/kg)	Head and neck Region (primary malignant neoplasm, recurrent tumour disease, lymph node metastasis)	squamous cell carcinoma, adeno- carcinoma, oropharynx, oral cavity, larynx, epipharynx, hypopharynx	3T	Switzer- land	Positive If their SUVmax was at least two-fold higher than surrounding background activity
Reinert et al. [38].	2022	[18F] Fluoride Positron-Emission Tomography (PET) and [18F]FDG PET for Assessment of Osteomyelitis of the Jaw in Comparison to Computed Tomography (CT) and Magnetic Resonance Imaging (MRI): A Prospective PET/CT and PET/MRI Pilot Study	J Clin Med	55.3 ± 10.0 yrs	Not Mentioned	6 F	6 participants	Biograph mCT Siemens	Biograph mMR Siemens Healthcare	Gadovist, Bayer Vital	Osteomye- litis	OMJ	Not men- tioned	Germany	healthy jawbone SUV_mean_ 15.4 ± 4.2, [18F] FDG uptake was moderately higher SUV_mean_ 1.9 ± 0.7)
Park et al. [39]	2020	Diagnostic Accuracy and Confidence of [18F] FDG PET/MRI in comparison with PET or MRI alone in Head and Neck Cancer	Scientific Reports	18–83 yrs	Not Mentioned	71 M 37 F	108 participants	NA	Biograph mMR Siemens Healthcare	Dotarem (0.1 mmol/kg)	Head and neck region (primary malignant neoplasm, recurrent tumour disease, lymph node metastasis)	pharynx, oral cavity, sinonasal cavity, parotid gland, larynx, infrate- mporal fossa, squamous cell carcinoma, lymphoma, adeno- carcinoma	3 T	Korea	Definitely benign SUV < 2.5, Score of 2 probably benign: SUV ≥ 2.5 Score of 4, probably malignant (2.5 ≤ SUV < 5) Score of 5 definitely malignant SUV ≥ 5

**Table 2:** Risk of bias of included non-randomized studies according to ROBINS-I tool.

Bias Due to								
	Confounding	Selection of Participants for the Study	Classification of Interventions	Deviations from Intended Interventions	Missing Data	Measurement of Outcomes	Selection of the Reported Result	Overall
Hayashi et al. 2019 [42]	Low	Moderate	Low	Moderate	Moderate	Moderate	Moderate	Moderate
Kanno et al. 2020 [43]	Low	Moderate	Moderate	Moderate	Moderate	Moderate	Low	Moderate
Loeffelbein et al. 2014 [44]	Low	Moderate	Low	Moderate	Low	Low	Low	Moderate
Schaarschmidt et al. 2015 [45]	Low	Serious	Serious	Moderate	Low	Moderate	Moderate	Serious
Samołyk -Kogaczewska et al. 2019 [46]	Low	Moderate	Low	Serious	Moderate	Moderate	Moderate	Serious
Tsujikawa et al. 2020 [47]	Low	Moderate	Moderate	Low	Low	Low	Low	Moderate
Samołyk -Kogaczewska et al. 2020 [48]	Low	Moderate	Moderate	Moderate	Low	Low	Moderate	Moderate
Freihat et al. 2021 [31]	Low	Moderate	Moderate	Low	Moderate	Low	Low	Moderate
Schaarschmidt et al. 2017 [40]	Low	Low	Moderate	Low	Low	Moderate	Moderate	Moderate
Queiroz et al. 2014 [41]	Low	Moderate	Low	Low	Low	Low	Moderate	Moderate
Park et al. 2020 [39]	Low	Moderate	Low	Low	Low	Low	Moderate	Moderate
Reinert et al. 2022 [38]	Low	Moderate	Low	Low	Low	Low	Moderate	Moderate
